# Multi-night acoustic stimulation is associated with better sleep, amyloid dynamics, and memory in older adults with cognitive impairment

**DOI:** 10.1007/s11357-024-01195-z

**Published:** 2024-05-14

**Authors:** Céline J. Zeller, Marina Wunderlin, Korian Wicki, Charlotte E. Teunissen, Christoph Nissen, Marc A. Züst, Stefan Klöppel

**Affiliations:** 1 University Hospital of Old Age Psychiatry and Psychotherapy, University of Bern, 3000 Bern 60, Switzerland; 2 Graduate School for Health Sciences, University of Bern, 3012 Bern, Switzerland; 3 Neurochemistry Laboratory, Department of Clinical Chemistry, Amsterdam Neuroscience, Amsterdam UMC, Vrije Universiteit Amsterdam, 1081 HV Amsterdam, Netherlands; 4 Division of Psychiatric Specialties, Department of Psychiatry, Geneva University Hospitals (HUG), 1201 Geneva, Switzerland; 5 Department of Psychiatry, University of Geneva, 1201 Geneva, Switzerland

**Keywords:** Sleep, Dementia, Prevention, Phase-locked auditory stimulation, Amyloid beta, Cognitive decline

## Abstract

Sleep is a potential early, modifiable risk factor for cognitive decline and dementia. Impaired slow wave sleep (SWS) is pronounced in individuals with cognitive impairment (CI). Cognitive decline and impairments of SWS are bi-directionally linked in a vicious cycle. SWS can be enhanced non-invasively using phase-locked acoustic stimulation (PLAS), potentially breaking this vicious cycle. Eighteen healthy older adults (HC, age_mean±sd_, 68.3 ± 5.1) and 16 older adults (age_mean±sd_, 71.9 ± 3.9) with CI (Montreal Cognitive Assessment ≤ 25) underwent one baseline (sham-PLAS) night and three consecutive stimulation nights (real-PLAS). EEG responses and blood-plasma amyloid beta Aβ42/Aβ40 ratio were measured pre- and post-intervention, as was episodic memory. The latter was again evaluated 1 week and 3 months after the intervention. In both groups, PLAS induced a significant electrophysiological response in both voltage- and time–frequency analyses, and memory performance improved in association with the magnitude of this response. In the CI group, both electrophysiological and associated memory effects were delayed compared to the healthy group. After 3 intervention nights, electrophysiological response to PLAS was no longer different between CI and HC groups. Only in the CI sample, stronger electrophysiological responses were significantly associated with improving post-intervention Aβ42/Aβ40 ratios. PLAS seems to improve SWS electrophysiology, memory, and amyloid dynamics in older adults with CI. However, effects on memory require more time to unfold compared to healthy older adults. This indicates that PLAS may become a potential tool to ameliorate cognitive decline, but longer interventions are necessary to compensate for declining brain integrity. This study was pre-registered (clinicaltrials.gov: NCT04277104).

**Supplementary Information:**

The online version contains supplementary material available at 10.1007/s11357-024-01195-z.

## Introduction

As we get older, sleep tends to worsen [1]. Impaired sleep has many negative health consequences [2, 3] and contributes to a deterioration in neurocognitive domains such as attention and memory and is overall associated with an increased risk of developing dementia [4–6]. Older adults with sleep problems are more likely to develop mild cognitive impairment (MCI) and Alzheimer’s disease (AD) [7, 8].

Impaired slow-wave sleep (SWS, the deepest sleep stage) affects up to two-thirds of individuals with dementia [9]. Electrophysiologically, SWS is characterized by the prevalence of slow wave- (~ 1 Hz), delta- (1–4 Hz), and sleep spindle activity (12–16 Hz; [10–12]. The interplay of these oscillatory components during SWS is important for memory consolidation [13, 14]. In older adults (especially with MCI or AD), slow wave (SW) activity significantly decreases, exhibiting lower amplitudes, shallower slopes, and fewer overall wave events [15, 16].

Amyloid-beta (Aβ) can be a potential mediator of the link between SWS and cognitive decline. Aβ plaques and neurofibrillary tangles are biomarkers of AD [17–22] and contribute significantly to the development of sleep problems during both the preclinical and clinical stages of AD. A single night of SWS deprivation can significantly increase the Aβ burden in the brain (which cannot be compensated in subsequent nights [23]). Reversely, Aβ has been shown to disrupt SWS [23–26]. This bi-directional connection results in a self-sustaining vicious cycle where less SWS increases Aβ, and more Aβ interrupts SWS [21]. Because both the accumulation of Aβ starts up to 20 years before the onset of cognitive symptoms [27, 28] and impaired SWS also commonly starts long before cognitive symptoms can be recognized [29, 30], early intervention is crucial to prevent this vicious cycle.

Recently, phase-locked acoustic stimulation (PLAS) during sleep has been discussed as a promising, non-invasive intervention to enhance SW activity. PLAS algorithms detect slow waves (SWs) and synchronize the presentation of acoustic stimuli to the peak of naturally occurring SWs, which induces more SW activity—acting like a pacemaker [31]. In younger adults, PLAS can enhance SW activity and lead to downstream memory improvements after 1 night [32]. In older individuals, these effects are not reliably seen after one stimulation session—multiple stimulation nights seem to be required to compensate for age-related reductions in SWS and memory performance, as we showed in previous studies [33–37].

Here, we set out to investigate the feasibility and efficacy of PLAS in a predementia sample of older individuals with cognitive impairment, compared to a healthy group, to investigate if PLAS can improve SWS (specifically increase slow wave-, delta-, and sleep spindle activity) in this sample and if there are effects on memory or Aβ ratio changes. Our assumption was that (a) there would still be sufficiently many SW events to induce significant PLAS effects in the EEG (based on previous research; Wunderlin et al., 2023, 2024) in a sample of older adults with cognitive impairments and (b.) it is still possible to improve memory and Aβ clearance with PLAS.

## Methods

### Participants

Participants were recruited by advertisements in regional newspapers. Inclusion criteria were age between 60 and 80 years, native (or comparably fluent) German speakers without dementia, normal or corrected-to-normal vision, and unimpaired hearing. Participants were divided into two groups based on their performance on the Montreal Cognitive Assessment (MoCA) [38, 39]. Participants with a MoCA score ≤ 25 were allocated to the cognitively impaired (CI) group, and those with a score > 25 to the healthy group (HC) [38]. In addition to showing suboptimal MoCA scores, individuals in the CI group all reported subjective cognitive decline and concerns about it.

Exclusion criteria were impaired hearing and sleep disorders assessed via the Berlin questionnaire [40] and the Regensburg insomnia scale [41]. Individuals with irregular sleep patterns, pre-existing neurological or psychiatric conditions such as depression assessed via the geriatric depression scale [42], and the intake of psychotropic drugs or sleep-dependent medication were excluded as well. A telephone screening and an adaptation/screening night in the sleep laboratory at UPD Bern were conducted to ensure suitability. To this end, participants were screened by a trained sleep rater for sleep quality–based exclusion criteria, like restless legs syndrome, sleep apnea, and sleep-related respiratory issues.

Participants completed questionnaires to evaluate their chronotype using the morningness-eveningness-questionnaire [43], face recognition ability using the P-20 questionnaire [44], quality-of-life measures using the SF-36 questionnaire [45], and self-reported sleep quality levels assessed via the Pittsburgh Sleep Quality Index [46]. All participants gave their written informed consent before participation. The study was approved by the ethics committee, and an incentive of 400 CHF was given to participants for completing the study.

We included 34 older adults in this study (age [*M* ± SD] = 70.02 ± 4.91; 20 female). Sixteen older adults (6 female) were allocated to the CI group, and 18 older adults (14 female) in the healthy group. Data from 18 participants have previously been reported [36]. Our study size is comparable with previous studies [31, 36, 37], whereas our CI group is almost double the size of previous research with amnestic MCI [47].

The general characteristics and sleep architecture of the participants are displayed in Tables 1 and 2. The group allocation was not balanced in relation to gender (*χ*^2^(1, *N* = 34) = 4.13, *p* = 0.042). The healthy group scored significantly higher on the MoCA (healthy [*M* ± SD], 27.94 ± 1.43; CI, 23.25 ± 1.57; *t*(32) = 9.112, *p* =  < 0.001) and was significantly younger than the CI group (healthy, 68.33 ± 5.13; CI, 71.94 ± 3.99; *t*(32) =  − 2.264, *p* = 0.03). Thus, we included gender and age as covariates in our main analyses, i.e., regressions of PLAS-induced physiological response on memory performance and blood plasma Aβ levels (see Online Resource 1).

**Table 1:** Characteristics of participants

	HC group		CI group		
	*M*	SD	*M*	SD	*p*
Male	4		10		
Female	14		6		.04
MoCA score	27.94	1.43	23.25	1.57	< .001
Age	68.33	5.13	71.94	3.99	.03
Education	3.22	1.06	3.31	0.95	.8
P-20	40.11	14.5	40.19	13.93	.99
SF-36 PH	51.44	5.96	51.13	3.13	.85
SF-36 MH	55.1	4.92	54.36	7.11	.72
AHI	8.3	7.51	9.37	9.83	.73
PSQI	3.83	2.04	5.125	3.01	.17
Baseline plasma Aβ42/Aβ40	0.069	.010	.070	.006	.73

The mean and standard deviation of relevant parameters are provided for both groups (*HC* healthy, *CI* cognitively impaired), except for gender variables, where counts are provided. An *X*^2^*-*test was used to compare the gender of the two groups, and* t*-tests were used for other parameters. Education was operationalized as a categorial variable expressing the highest educational level achieved (from 1 = primary school to 4 = university). SF-36 PH and SF-36 MH are quality-of-life measures focusing on physical health and mental health, respectively, ranging from 0 (very bad) to 100 (perfect quality of life). The Apnoea-Hypopnoea-Index (AHI) was used to exclude sleep apnea, and the Pittsburgh Sleep Quality Index (PSQI) was used to measure subjective sleep quality (a lower score stands for better subjective sleep quality). For baseline plasma Aβ42/Aβ40, lower ratios indicate a higher risk of developing AD

**Table 2:** Changes in sleep architecture in the HC and CI groups

	BL		E1		E2		E3	
	CI	HC	CI	HC	CI	HC	CI	HC
N1	109.310	91.970	109.750	88.777	105.406	89.833	98.687	91.166
N2	170.343	154.944	163	183.277	178.375	167.527	188.093	177.305
N3	18.8125	38.4444	19.281	32.333	18.968	40.583	16.437	40.611
REM	44.625	45.666	47.7812	61.861	49.718	60.611	55.437	65.277
W	148.531	128.222	147.968	118.694	119.687	106.638	129.906	108.111
TST	343.093	331.027	339.812	366.250	352.468	358.555	358.656	374.361
SL	13.2187	17.805	17.375	14.861	11.156	16.777	11.093	17.027
SE	70.219	73.399	69.755	75.701	75.032	77.454	73.576	77.902
WASO	135.968	110.916	131.093	104.333	109.031	90.361	119.312	91.583

Mean values of the sleep architectural parameters per night (baseline (BL) and experimental nights E1–E3) and per group (cognitively impaired (CI) and healthy (HC)). Sleep stages N1, N2, N3. and REM as well as wake time (W) and sleep latency (SL) are expressed in minutes

*TST* total sleep time (in minutes), *SE* sleep efficiency (percentage of sleep relative to the minutes spent in bed), *WASO* wake time after sleep onset (in minutes)

### Study design and material

**Fig. 1:**
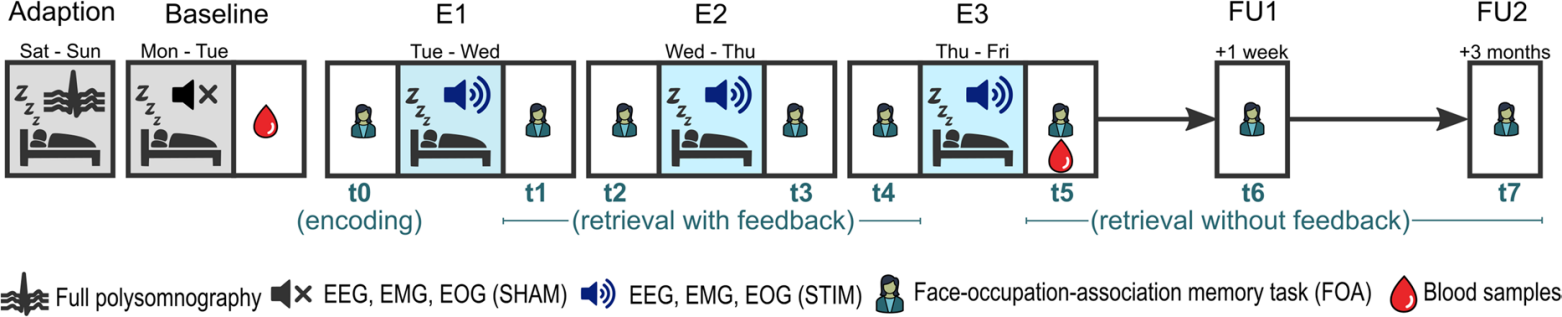
Study procedure. All participants of this study (healthy control, HC, and cognitively impaired (CI)) spent 5 nights in the sleep laboratory including a phase-locked auditory stimulation (PLAS) paradigm. First, an adaptation night served as a screening night for sleep pathologies, with full polysomnography (including leg electromyography, chest and abdominal belts, airflow sensors, and oximetry), but no PLAS was applied. After 1 night at home, participants slept in the sleep laboratory for 4 consecutive nights (a baseline night and 3 experimental nights, E1 through E3). In the morning after the baseline (sham-PLAS) night, a pre-intervention blood sample was collected. During the 3 experimental nights, real-PLAS was applied. Every evening and morning, participants performed a face-occupation-association memory task (FOA task, t0–t5), with t0 serving as the baseline assessment before the stimulation nights. In the morning after the third experimental night, a post-intervention blood sample was collected. As follow-ups (FU), the FOA task was reassessed at 1 week (FU1, t6) and 3 months (FU2, t7) after the intervention

Participants spent 5 nights in the sleep laboratory at UPD Bern (see Fig. 1): an adaptation/screening night (without PLAS), a baseline night (with sham-PLAS), and 3 experimental nights (with real-PLAS). During the adaptation/screening night, full polysomnography was recorded to screen for potential sleep-based exclusion criteria. After the adaptation/screening night, participants spent 1 recovery night at home. The remaining 4 nights followed consecutively. In the evening of the baseline night, a hearing test was completed where PLAS volume was set to the individual hearing threshold plus a fixed volume, resulting in an average PLAS sound pressure level of 50 dB as measured at the headbands’ integrated speakers. Before each experimental night, the calibrated presentation volume was retested and recalibrated if necessary.

During all nights in the laboratory, participants wore a 128-channel MicroCel Geodesic Sensor net (400 series Geodesic EEG System™, Electrical Geodesics, Inc.) recording Electroencephalography (EEG), a headband with speakers for the stimulation (sleepphones®, AcousticSheep LLC), and a two-electrode electrocardiogram (ECG). All measurements except the EEG were recorded on a Physio16 input box (Electrical Geodesics, Inc. EGI, Eugene, OR, USA).

Participants were instructed to follow their regular circadian rhythm and keep it constant over the 5 nights. In the evening and in the morning of each night, participants completed the Stanford Sleepiness Scale [48], the Tiredness Symptoms Scale [49], and a sleep diary [50].

#### Sleep scoring and acoustic stimulation

A sleep rater scored sleep stages according to the criteria by the American Academy of Sleep Medicine [51] for all recorded nights.

To detect SWs online and precisely apply sound presentations phase-locked to the peak of SWs, we used a template-based algorithm described elsewhere in more detail [52, 53]. In brief, the online algorithm analyzes rising voltage in frontal channels and computes topographic correlation with a canonical template map of a SW-peak within the most recent 120 ms of data. If both voltage and correlation with the topographic template are clearly rising during this 120 ms (specifically, if the average of the sign of the first derivative of both voltage and correlation to the topographic template across the 120 ms of data were larger than 0.75), a peak prediction is scored. During real-PLAS, a 50-ms pink noise sound is then presented after a delay calibrated to coincide with the predicted SW peak (typically ~ 50 ms). During sham-PLAS (baseline night), sham markers were set by the algorithm but no sound was played.

#### Amyloid beta (Aβ)

In the morning, after the baseline night (pre-intervention) and the third experimental night (post-intervention, PI), blood samples were collected, instantaneously centrifuged, and stored at − 80 °C. The samples were sent to the Amsterdam University Medical Center (NL) for analysis. Using N4PE Simoa immunoassays (IA-N4PE) plasma-amyloid beta 1–42 (Aβ42) and 1–40 (Aβ40) isoforms were quantified (commercially available from Quanterix, Billerica, Massachusetts; [54, 55]). Lower Aβ42/Aβ40 ratios in the blood are associated with a higher risk of developing MCI or AD [56]. Aβ42/Aβ40 ratios were calculated pre- and post-intervention. To analyze plasma Aβ42/Aβ40 response to treatment, a difference score (from pre- to post-intervention) was calculated. A more positive difference score is indicative of a beneficial response to treatment in Aβ dynamics due to more Aβ being removed from the brain and transported into the bloodstream [57].

#### Face-occupation associations (FOA) task

The face-occupation association task (FOA task) [36, 37] assesses cumulative (repeated) hippocampus-dependent episodic memory performance, allowing the repeated observation of the same memory traces across multiple sessions. The FOA stimuli consisted of 20 female and 20 male faces, which were randomly paired with 20 occupations (each occupation was repeated once as female and once as male variant). The faces were selected from a database of artificially created faces using generative adversarial networks [58]. They were comparable in terms of perceived age, income, attractiveness, and recognizability [36]. Each participant was assigned an individual and random association of the 40 faces with the 20 occupations, resulting in a unique set of FOA stimuli per participant.

**Fig. 2:**
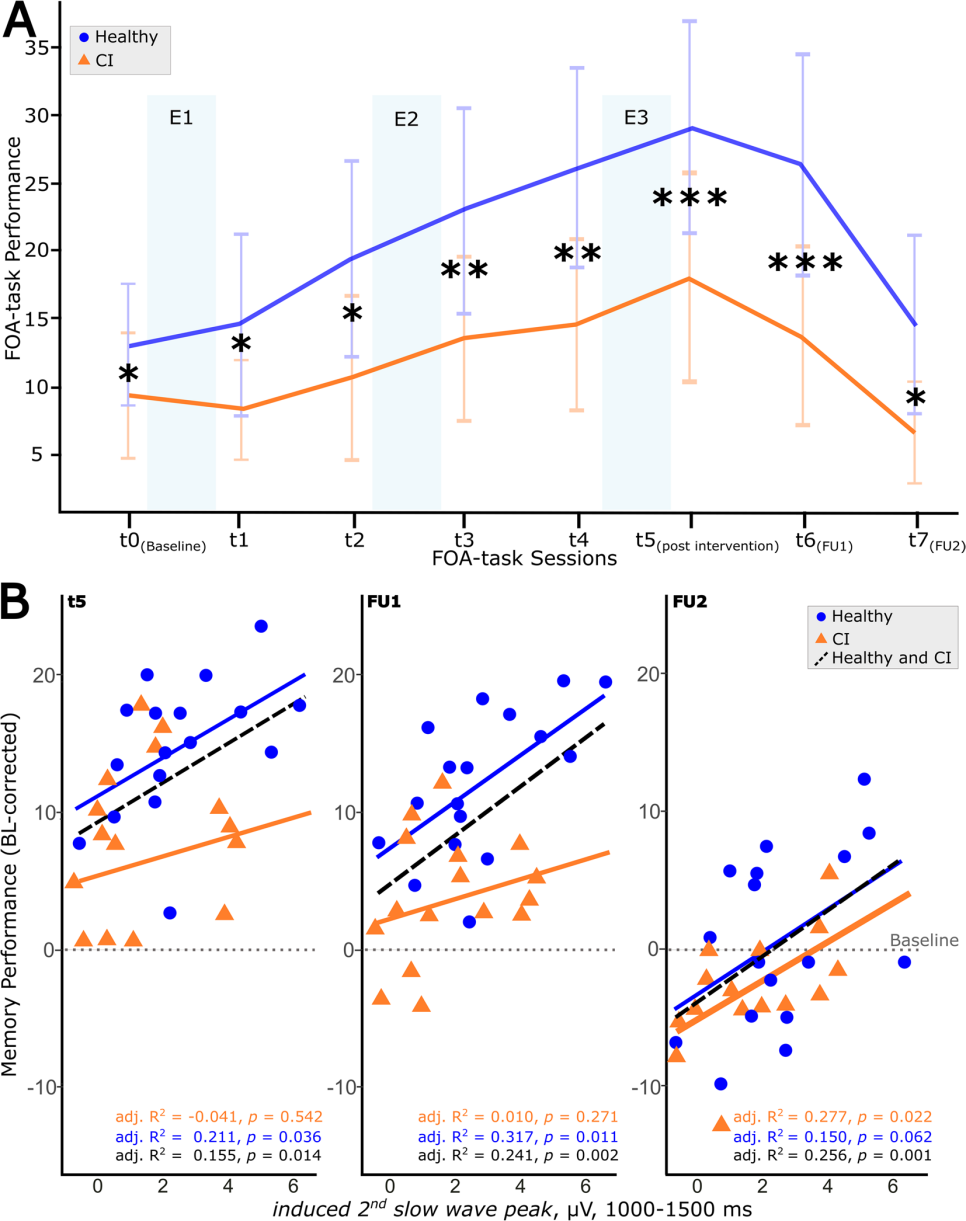
Memory performance across the intervention period. **A** The progression of the mean (± SD) memory performance of the healthy (in blue) and cognitively impaired (CI, in orange) groups for each time point of the face-occupation association (FOA)-task (t0 = baseline, t1–t4 = during intervention, t5 = post-intervention, t6 = 1-week follow-up (FU1), t7 = 3-month follow-up (FU2)). The light blue bars behind the curves indicate when experimental nights (E1–E3) occurred. The healthy group performed significantly better in this task compared to the CI group at all time points (*p < .05; **p < .01; ***p < .001). **B** Electrophysiological response to PLAS (magnitude of induced second slow-wave peak) predicts FOA memory performance (baseline-corrected) after 3 nights of intervention (post-intervention; t5), after 1 week (follow-up 1; FU1), and after 3 months (follow-up 2; FU2) for the whole sample (black dashed line). In the healthy group (blue line), t5 and FU1 exhibit a significant association with FU2 reaching trend level, and in the CI group (orange line), only FU2 exhibits a significant association, illustrating a delayed effect of PLAS on memory in cognitively impaired individuals. Including age and gender as covariates in these linear regression models did not meaningfully change the results (see Online Resource 1)

The FOA stimuli were presented for initial encoding during two runs on the evening before the first experimental night (see Fig. 1, t0 as baseline). Participants were instructed to focus on the screen and to memorize as many associations as possible. Stimuli were presented in a randomized order for each run. Faces were presented to the left and occupations to the right of a fixation cross. Each stimulus was presented for 5000 ms with an inter-stimulus interval of 500 ms (black screen). After the second encoding run, an immediate cued recall started. The faces were shown alone, one by one, in randomized order, and participants were instructed to verbally communicate the corresponding occupations at a self-paced speed. Responses were recorded and subsequently transcribed offline after the sessions. This immediate recall served as a baseline measure (see Fig. 2A, t0). Cued recall was tested each evening and morning throughout the experimental nights (t1–t5) and at the two follow-up measurements (t6, t7), with the faces being presented randomly each time. Visual feedback showing the correct answer was provided during all recall trials, except for the post-intervention session (t5) and both follow-up sessions (t6, t7). Feedback served as additional learning runs, gradually improving performance. The number of stimuli (40) was calibrated to allow the mapping of naturalistic learning curves, preventing ceiling effects post-intervention [36, 37]. To analyze memory gains, we subtracted performance at baseline (t0) from performance at each subsequent cued recall session (t1–t7).

### Statistical analysis

The data analysis was conducted using RStudio (version 22.07.1) available at https://dailies.rstudio.com/version/2022.07.1+554/, and MATLAB (version R2022b, The MathWorks, Natick, Massachusetts) using the Toolboxes FieldTrip [59] and EEGLAB [60]. The raw EEG data was down-sampled to 200 Hz and preprocessed with the PREP pipeline [61]. Fieldtrip’s automatic artifact rejection pipeline was used for detecting high-frequency (HF) noise, muscle artifacts, and signal jumps as well as bad channels. All EEG analyses were based on non-REM sleep stage 2 and SWS.

#### PLAS-induced electrophysiological responses based on event-related potentials and spectral perturbations

PLAS-induced electrophysiological responses were evaluated using event-related potentials (ERPs) and event-related spectral perturbations (ERSPs) for each group and each experimental night locked to PLAS markers. The ERPs were calculated by epoching the data around the stimuli between − 1.5 and 3 s and were baseline-corrected by subtracting the mean signal of the whole epoch from each time point. For statistical differences between the experimental and baseline nights, we used non-parametric permutation tests (*p* < 0.05) as implemented in the FieldTrip Toolbox [59].

**Fig. 3:**
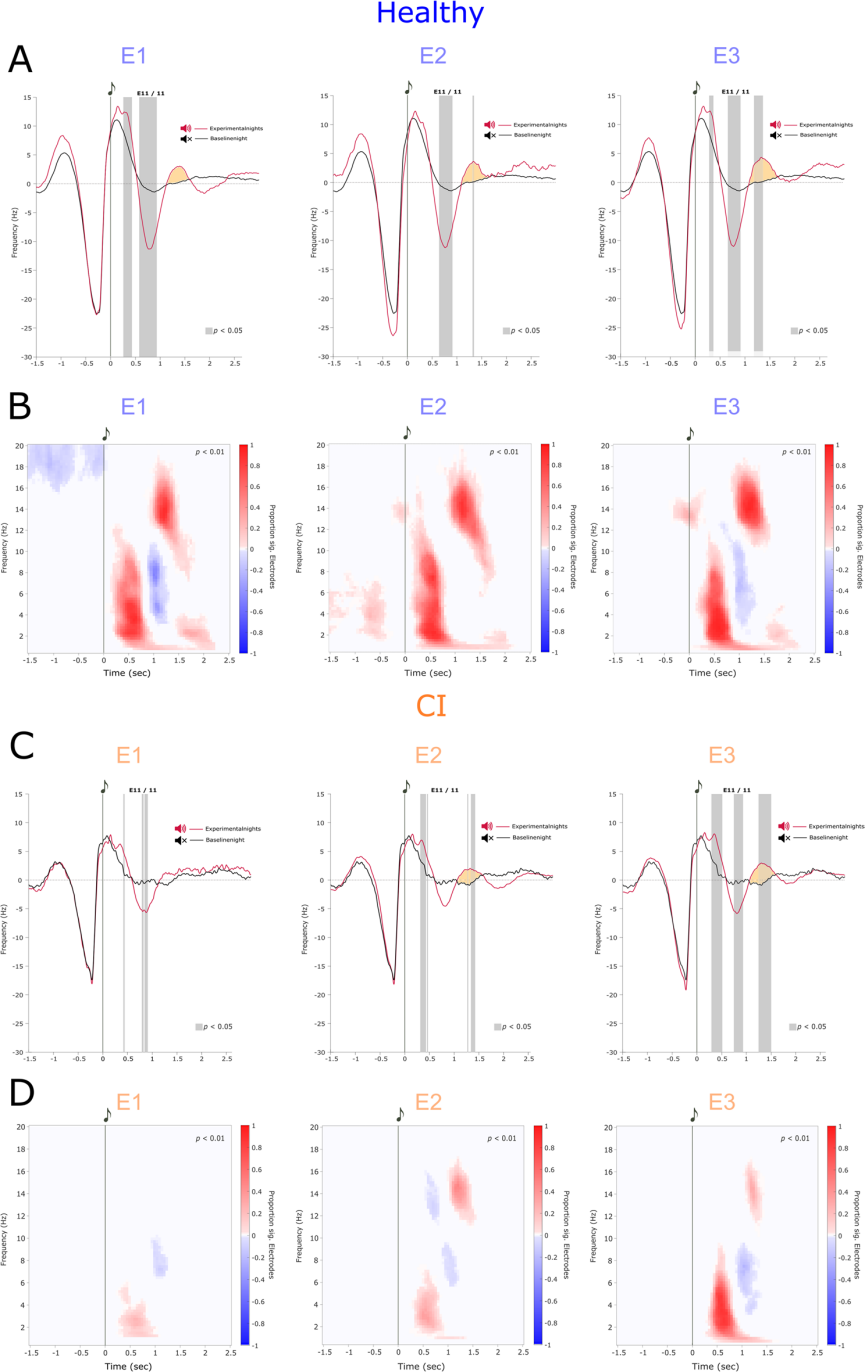
Cluster-based event-related potentials (ERP) and event-related spectral perturbations (ERSP)*.* **A** and **C** ERP of the healthy- and the cognitively impaired (CI) group in each experimental night (E1, E2, and E3). Significant clusters in the ERP (*p* < 0.05) are shown as gray bars behind the curves. The second induced slow wave (SW) peak is highlighted in orange for visual inspection. **B** and **D** Significant ERSP clusters of the healthy and CI groups in each experimental night (E1, E2, and E3) vs. the baseline night (*p* < 0.01). Positive clusters are shown in red (i.e., increased activity in experimental vs. baseline night), and negative clusters are in blue (i.e., decreased activity in experimental vs. baseline night)

To quantify the individual electrophysiological response to real-PLAS, we calculated weighted means of the voltage difference of experimental and baseline nights in electrode Fz at 1–1.5 s post-stimulus (where the occurrence of PLAS-induced peaks is expected and visually confirmed, see Fig. 3 and Wunderlin et al. (2023, 2024). To account for varying numbers of applied stimulations across experimental nights, we used the number of stimulations per experimental night as weights for the calculation of the average induced electrophysiological response [36, 37]. The weighted mean of the voltage difference between experimental nights and baseline nights (= individual electrophysiological response) was used in linear regression models to predict Aβ response and memory performance. To account for potential age and gender effects, we calculated additional linear regression models with these two covariates and compared the two models, with and without covariates.

Based on previous findings [36, 37] we analyzed spectral responses to PLAS. ERSPs were calculated using Morlet wavelet transforms. We focused on epochs of − 1.5 and 2.5 s for the frequency range between 0.5 and 20 Hz. The time window from 2 to 2.5 s exhibited the least amount of spectral activity and was therefore used as the baseline. For statistical differences between the experimental and baseline nights, we again used non-parametric permutation tests as implemented in the FieldTrip Toolbox [59]. To visualize the magnitude of spectral perturbations of experimental vs. baseline nights, we plotted the proportion of channels exhibiting a significant difference between baseline and experimental nights at each time–frequency bin (see clusters in Fig. 3B and D).

To test the individual electrophysiological response to real-PLAS in the time–frequency representation, power values from significant time–frequency bins were extracted. Analogously to the ERP-based electrophysiological response value, for specific time-electrode-frequency bands, we subtracted the median power in the baseline night from the median power in each experimental night and calculated weighted means using the number of stimulations per night as weights. The time-electrode-frequency bands were based on the timing of the induced peak and trough within the stimulation window (0.5–1 s, 1–5 s), across specific frequency bands (0.75–1.5 Hz = slow waves, 1–4 Hz = delta, 4–8 Hz = theta, 12–16 Hz = spindle, 16–20 Hz = beta) and electrodes (13 centroparietal electrodes centered around Cz for spindle power and 17 frontal electrodes around Fz for all other bands). The weighted mean of these power differences between experimental nights and baseline nights (= individual electrophysiological response) was again used in linear regression models to predict Aβ response and memory performance.

Finally, to test whether the electrophysiological response to PLAS is consistent across all nights or whether on the contrary there are potential linear increasing/decreasing trends, we calculated the absolute summed activity of each experimental night relative to the baseline night in the spectral response. To achieve this, we first masked each participant’s time–frequency power matrices (from the ERSP analyses) by the group-level significant clusters, per experimental night. We then further restricted the resulting mask to the post-stimulation time window (0–2.5 s) and frequencies between 0 and 16 Hz. Next, we extracted the power (in dB) of the experimental- divided by baseline nights, as absolute values (negative and positive clusters valued the same), of each time–frequency bin in the mask. After we excluded bins with division results > 1.5 SD away from the individual mean and summed up all remaining bins per experimental night. This yielded a value, per participant and experimental night, of the summed, PLAS-evoked activity, capturing both the height (peak differences) and width (cluster extents) of induced spectral activity. Next, for each experimental group, we calculated linear regression analyses using consecutive experimental nights as predictors for the summed activity, to test for linear trends in electrophysiological response across the intervention period. Additionally, we tested the summed activity of each experimental night between groups using independent *t*-tests.

## Results

### Sleep architecture

No baseline values of the sleep architecture differed significantly between the two groups. Difference scores as measures for changes in sleep architecture between the two groups were calculated by collapsing all experimental nights and subtracting the baseline night’s value. A Holm–Bonferroni-corrected *t*-test between the CI and healthy group was calculated for all difference scores. The difference scores were further tested against zero within the groups (Holm–Bonferroni corrected) to determine whether there were differences between the experimental and the baseline nights. The time spent in each sleep stage, total sleep time (TST), the proportion of sleep in relation to time spent in bed (sleep efficiency, SE), time spent awake after first falling asleep (WASO), and subjective sleep quality are summarized in Table 2. None of the difference scores (experimental–baseline) statistically differed from zero in either group, and no sleep characteristics differed significantly between the two groups (all adjusted *p* > 0.5).

### Electrophysiological response to PLAS: induced second slow-wave peak

Figure 3A and C shows the ERP of the baseline night (black line) and all experimental nights (red lines) with an induced trough around 0.5–1 s and, indeed, an induced second SW peak around 1–1.5 s after the stimulation (0 s) for the healthy group (Fig. 3A) and the CI group (Fig. 3C). The gray bars behind the curves in the ERPs of Fig. 3 show clusters of significant difference between experimental and baseline nights (*p* < 0.05). These time windows represent the electrophysiological response to PLAS. Visually, the electrophysiological response seems to get stronger (i.e., larger differences, wider and more numerous clusters of difference in gray) across the experimental nights in the CI group but stays stable over the three experimental nights in the healthy group. For a formalized analysis of this apparent development, see below (section “Delayed electrophysiological effect in CI group”).

### PLAS-induced electrophysiological response predicts amyloid beta (Aβ) response to treatment

**Fig. 4:**
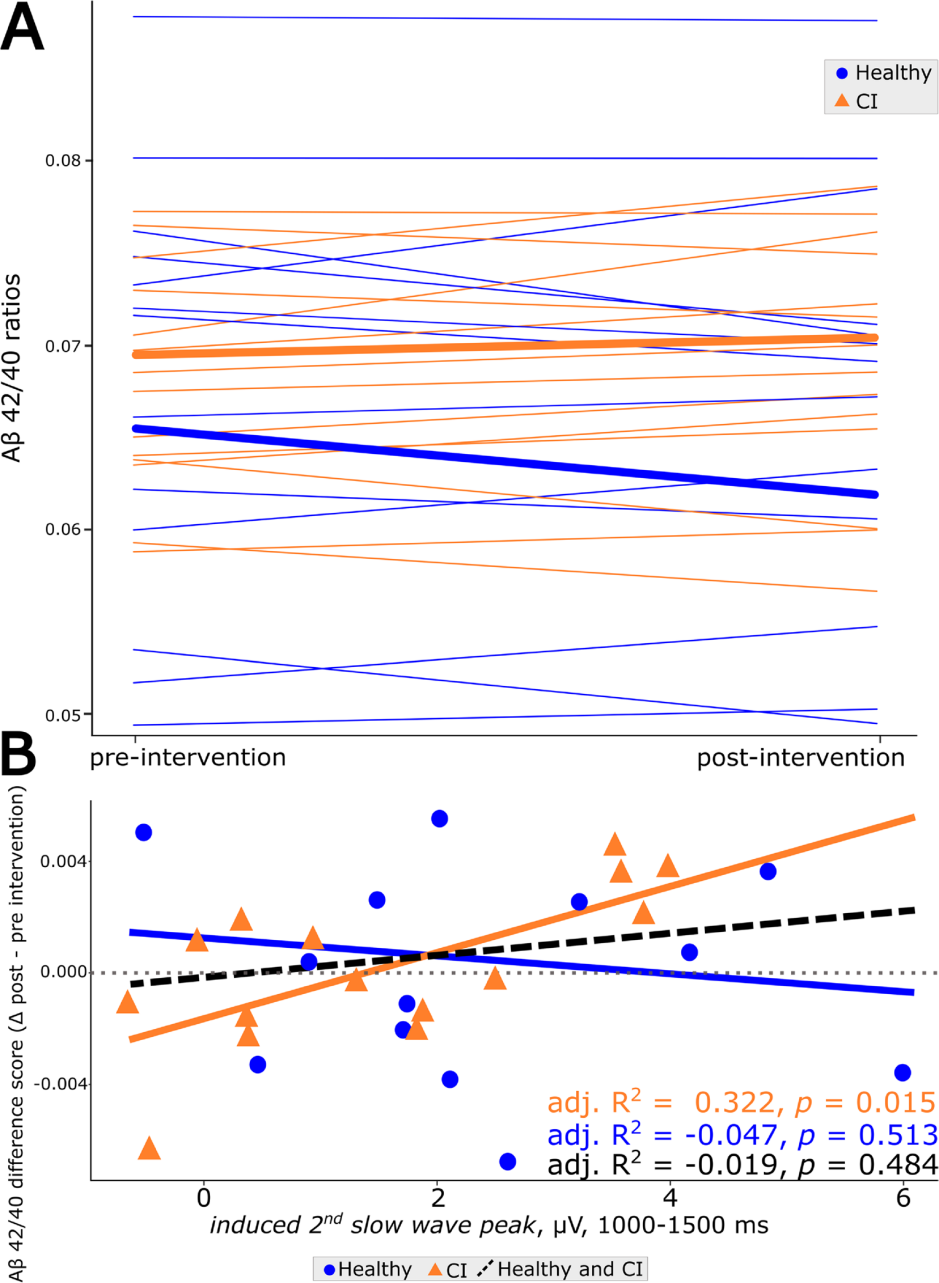
Electrophysiological response predicts amyloid-beta 42/40 improvement. **A** Spaghetti plot of each participant’s (healthy in blue, CI in orange) Aβ42/Aβ40 ratio at baseline (pre-intervention) and after the intervention of 3-night PLAS (post-intervention). **B** Linear regressions with Aβ42/Aβ40 change scores (post- to pre-intervention, where a higher score represents a beneficial development) and the induced second slow-wave peak were calculated. The black line is the overall intervention effect for all stimulated participants, the blue line is the linear regression of the healthy group alone, and the orange line is the linear regression of the CI group alone. Effect sizes are indicated using adjusted *R*.^2^ values. Including age and gender as covariates in these linear regression models did not meaningfully change the results (see Online Resource 1)

To investigate the effect of PLAS on Aβ response to treatment, we regressed PLAS-induced electrophysiological response (i.e., the magnitude, or averaged voltage, of the induced second SW peak) onto the Aβ difference score (Aβ 42/40 ratio post–pre intervention). While no significant effect occurred across all 34 participants (F(1,26) = 0.50, adj*. R*^*2*^ =  − 0.02, *p* = 0.48), nor in healthy participants (F(1,11) = 0.46, adj*. R*^*2*^ =  − 0.05, *p* = 0.51), a stronger induced second SW peak correlated with a beneficial Aβ42/Aβ40 ratio change in the CI group (F(1,13) = 7.66, adj*. R*^*2*^ = 0.32, *p* = 0.02, Fig. 4).

Importantly, Aβ42/Aβ40 ratios at baseline (pre-stimulation) did not significantly predict the induced second SW peak in either group, indicating that the change in amyloid dynamics from pre- to post-intervention was specific to PLAS.

### Delayed memory effect in the CI group

Performance in the FOA task before the first experimental night (see Fig. 2A, t0) served as a baseline memory assessment. At baseline, the healthy group performed significantly better than the CI group (healthy [*M* ± SD], 13.11 ± 4.43; CI, 9.38 ± 4.70, *t*(32) = 2.37, *p* = 0.012). This pattern continued throughout the intervention: In the morning after the third experimental night (E3, healthy, 29.05 ± 5.67; CI, 18.01 ± 5.90, *t*(32) = 3.68, *p* < 0. 001), at the 1-week follow-up (FU1, healthy, 26.39 ± 5.92; CI, 13.69 ± 4.70, *t*(32) = 3.13, *p* < 0.001), and the 3-month follow-up (FU2, healthy, 14.52 ± 6.88; CI, 6.56 ± 4.23, *t*(32) = 3.13, *p* = 0.04), the healthy group outperformed the CI group (Fig. 2A).

To investigate the downstream effect of PLAS on memory, we regressed PLAS-induced electrophysiological response (i.e., the magnitude of the induced second SW peak) onto memory performance at t5 (post-intervention) and at the two follow-ups (FUs, Fig. 2B). We compared these regression models with regression models including age and gender as covariates (see Online Resource 1). Electrophysiological response predicted memory post-intervention (Ft5(1,31) = 6.72, adj. *R*^2^ = 0.15, *p* = 0.01), and this relationship was stable at the 1-week (FFU1(1,31) = 11.42, adj. *R*^2^ = 0.24, *p* = 0.002) and 3-month follow-ups (FFU2(1,31) = 12.34, adj. *R*^2^ = 0.26, *p* = 0.001) across all 34 participants (both groups together).

In the healthy group, electrophysiological response predicted memory post-intervention (Ft5(1,15) = 5.286, adj. *R*^2^ = 0.211, *p* = 0.03) and in the 1-week follow-up (FFU1(1,15) = 8.410, adj. *R*^2^ = 0.316, *p* = 0.01) but reverted to trend-level at the 3-month follow-up (FFU2(1,15) = 3.81, adj. *R*^2^FU2 = 0.14, *p*FU2 = 0.07).

In the CI group, electrophysiological response initially (i.e., post-intervention and at the 1-week follow-up) did not predict memory (Ft5(1,14) = 0.41, adj. *R*^2^ =  − 0.04, *p* = 0.53, FFU1 (1,14) = 1.15, adj. *R*^2^ = 0.01, *p* = 0.30). Interestingly, however, at the 3-months follow-up, electrophysiological response significantly predicted memory (FFU2(1,14) = 6.75, adj. *R*^2^ = 0.27, *p* = 0.02), indicating a delayed effect in the CI group.

### Delayed electrophysiological effect in the CI group

Cluster-based significance testing of the ERSP in the experimental nights confirmed diverging patterns of the two groups: in the healthy group, there was a consistent, significant (*p* < 0.01) increase in power in the SW (~ 1 Hz), delta (1–4 Hz), and theta (4–8 Hz) ranges in all three experimental nights around the induced second SW peak (see Fig. 3B). In parallel, there was a consistent, but slightly shifted, decrease in theta and delta powers and an increase in spindle power (12–16 Hz) around the induced second SW peak as well as decreased beta power (12–16 Hz) in the pre-stimulus window. In the CI group, however, the magnitude of induced power changes seemed to increase across the three experimental nights in the same power frequencies (see Fig. 3D).

**Fig. 5:**
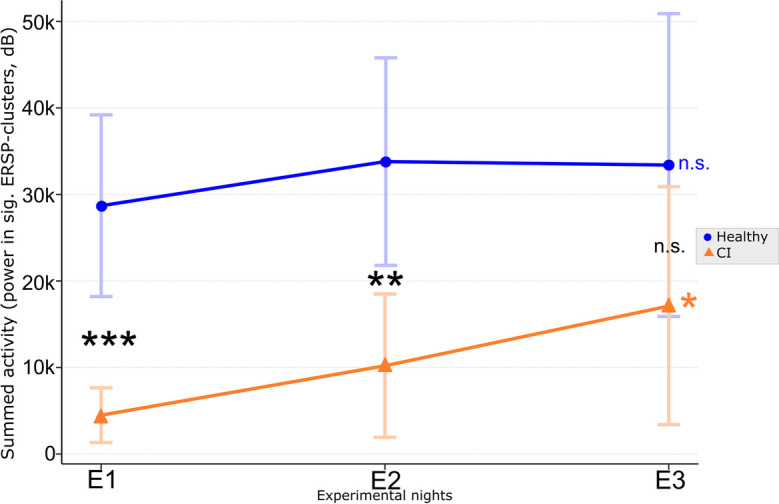
Summed power in significant ERSP clusters. The summed activity in the time (0–2.5 s) and frequency (0–16 Hz) range of interest was calculated within significant clusters from the ERSP analyses (cf. Figure 3). As a baseline correction, power values of each experimental night were divided by power values of the baseline night. Linear regression analyses revealed that for the CI group, summed activity increased across experimental nights E1 through E3 (orange asterisk), while summed activity stayed constant in the healthy group (blue “n.s.”). In addition, while nights E1 and E2 exhibited significant differences in summed activity between groups, this difference was no longer significant at night E3 (black asterisks). *p < .05; **p < .01; ***p < .001

To quantify this apparent divergence in the pattern of PLAS-induced activity, we summed the induced power (experimental/baseline) in the time–frequency representation of each experimental night and tested for linear trends across the consecutive experimental nights. The CI group showed a significant increase in the summed activity across experimental nights (*t*(16) = 2.08, adj.* R*^2^ = 0.06,* p* = 0.04) compared to the healthy group, which exhibited a constant summed activity across nights (*t*(18) = 0.51, *adj. R*^2^ =  − 0.01,* p* = 0.60, see Fig. 5). Next, we calculated independent *t*-tests for group differences of the summed activity in each experimental night. In nights E1 (*t*(32) = 4.42,* p* < 0.001) and E2 (*t*(32) = 3.33, *p* = 0.002), the healthy group exhibited significantly stronger summed activity compared to the CI group. In night E3, however, this difference was no longer significant (*t*(32) = 1.503,* p* = 0.142), indicating a delayed electrophysiological response in the CI group that eventually became comparable to that of the healthy group (see Fig. 5).

Finally, we wanted to test if PLAS-induced activity in specific frequency bands drove the effects on memory and plasma Aβ response. For this purpose, we extracted induced power in significant time–frequency clusters and regressed these spectral electrophysiological response values onto memory and Aβ change scores. No specific spectral response cluster alone consistently predicted Aβ response to treatment or memory performance (*p* > 0.08).

## Discussion

In this study, we show that enhancing sleep SW activity through a PLAS intervention across 3 consecutive nights has clear effects on sleep electrophysiology, inducing more slow waves and associated faster oscillations, including the delta, theta, and spindle bands. The magnitude of the induced SW activity is associated with improved Aβ dynamics and memory performance in older adults with cognitive impairment. We compared this population with a higher risk of developing dementia to a previously reported group of healthy older adults [36].

First, the CI group exhibits delayed electrophysiological responses to PLAS. In the healthy group, we see a strong and stable electrophysiological response to PLAS throughout all experimental nights, as previously reported [36, 37]. In the CI group, however, this electrophysiological response increases across the intervention period, starting rather weak on the first experimental night and only reaching a magnitude comparable to that of the healthy group on the third (last) experimental night Fig. 5).

Second, while our previous studies indicated that healthy older adults require more stimulation nights to benefit from PLAS comparably to what the literature indicates for younger adults [36, 37], the CI group seems only exhibit a consistent reaction reflected in a correlation between PLAS and memory performance months later. Arguably, this very delayed memory effect may be a different process taking hold than what drives memory gains during the intervention: while improved learning capacity may drive the increased gains during the intervention, it is probably long-term consolidation that establishes memory traces after 3 months [13]. PLAS could also have enhanced retrieval success, but this would arguably impact both early and late retrieval and is therefore a weaker candidate for being the driving factor here [13]. Hence, we argue that PLAS may have improved long-term consolidation, but not learning capacity or retrieval success, in the CI group (yet). Hypothetically, extending the intervention period across more nights might have allowed for delayed improvements of learning capacity or retrieval success to manifest in the CI group, paralleling the results of the healthy group.

Third, we found that plasma Aβ levels responded to treatment in the CI, but not in the healthy group. Notably, this interaction occurred in the absence of differences in the ratio between groups at baseline. The Aβ response was consistent with beneficial changes one would expect due to improved metabolic clearance: Plasma Aβ 42/40 ratios were increased in the blood after the intervention period, suggesting Aβ may have been removed from the brain and into the bloodstream [54, 55]. Previously, we reported a weak association between PLAS-related memory improvement and beneficial plasma Aβ change from pre- to post-intervention in a partly overlapping sample of healthy older adults [36]. Here, we see a more direct relation between instant electrophysiological response to PLAS and Aβ dynamics in a CI sample. Only seeing this relationship in the CI group is not unexpected, as their Aβ dynamics are potentially already suboptimal, with room for improvement. It has been suggested that improving Aβ dynamics may mitigate cognitive decline [62]. Thus, inducing more SW activity may convey multiple synergistic benefits at once: enhancing opportunities for memory reorganization to occur and improving Aβ dynamics, both benefiting cognitive performance.

Lastly, our findings highlight the need for long-term assessments—especially in older adults with cognitive decline. It seems the hypothesis that older brains might need more nights to react to the PLAS intervention holds even more strongly for older adults with CI. As individuals with cognitive impairment exhibit strongly reduced SWS [21, 30], there might not be enough remaining SW activity for the stimulation to engage with—at least at first. This indicates that “intact” SWS at baseline is needed for enhancing it, and this enhancement achieved with PLAS predicts the degree of cognitive improvement and Aβ response [63]. Only after longer intervention periods, the cumulative effect of repeated PLAS might become meaningful as we saw in the electrophysiological effects. Here, we did not see any indication that a plateau in the effectiveness of long-term PLAS interventions has been reached. Therefore, we argue that longer interventions are necessary to unlock the full potential of this intervention. As prolonged studies in a sleep laboratory quickly become ecologically and economically unfeasible, it might be advisable to employ portable solutions to move the intervention into the comfort of participants’ own home [64]. This transition opens new avenues for research and, ultimately, the implementation of practical preventive tools to challenge cognitive decline.

It is important to identify the target group for such interventions to initiate preventive measures before the onset of dementia to effectively prevent cognitive decline and its underlying factors, such as the accumulation of Aβ and memory loss. The predementia continuum between cognitively healthy and impaired seems to represent an opportunity where it is not too late to improve Aβ dynamics and/or memory functions with PLAS. Therefore, PLAS could be used as a preventative intervention method before diagnoses of dementia occur.

### Limitations

Although we did have a solid evaluation of the within-participant pre-intervention status, it is important to note that this study did not compare results to a CI group that performed the same behavioral tasks but only received sham stimulation across all nights. In previous studies, we did compare healthy older adults to such a control group, both between- [36] and within-subject [37] and confirmed effects on memory and Aβ dynamics were indeed PLAS-induced. Therefore, we may assume that the same is true here. Still, firmer conclusions would have been possible with the inclusion of a sham-only control group with CI.

Further limitations are related to the size but also the composition of the sample. We deliberately included participants on the border between intact and impaired cognition. Recruiting a sample of individuals with amnestic MCI may have led to clearer signals in Aβ dynamics but with a greater risk of too little SW activity to induce a significant electrophysiological response. Gender and age were not balanced across the two groups, with the CI participants being mostly male and on average 3–4 years older. However, including age and gender as covariates in our regression models did not change the main results (see Online Resource 1), and since our key findings were all related to within-group effects, these imbalances are unlikely to be a confounding factor.

We argue that one explanation for the delayed effects of PLAS on memory in the CI group may be that different memory processes were at play (i.e., new learning vs. retrieval success vs. long-term consolidation). However, our memory task was designed to observe single memory traces across the intervention period to investigate the cumulative effects of PLAS. Therefore, it is not optimized to distinguish improved learning capacity, retrieval success, and long-term consolidation, leaving this interpretation to speculation.

A further limitation is related to the interpretation of the Aβ changes in the blood given that this technique is still being established. We tentatively hypothesized that the effect presented here is consistent with improved clearance since this interpretation is supported by previous reports linking metabolic clearance to SW sleep [21]. Fundamentally, however, this interpretation is speculative until direct comparative studies can close this gap. Animal studies could directly relate PLAS-induced changes in Aβ dynamics in the blood plasma with Aβ dynamics in the brain.

Finally, we currently do not know all the implications of prolonged PLAS interventions. As we suggest longer intervention periods are needed, we must pay close attention to potential side effects, like impoverished quality of life and sleep. However, a recent study utilizing prolonged PLAS in older adults in an at-home setting did not report any indications of deleterious side effects [65].

### Conclusion

With the present study, it is encouraging to see that a non-invasive intervention such as PLAS can engender significant (delayed) benefits on a physiological (including Aβ) and behavioral level in older adults with CI. Thus, further investigation is crucial to uncover the underlying mechanisms by which PLAS influences memory consolidation and the clearance of Aβ in the target population. This study contributes to the existing literature on the use of PLAS as a preventive measure against cognitive decline by providing further insights into its effects in a specific predementia population. Enhancing SWS could be a promising strategy to prevent cognitive decline, potentially facilitated using portable auditory stimulation devices [64]. Such insights will facilitate the development and implementation of effective and efficient preventive tools. PLAS could become a non-invasive home-based tool to improve SWS and benefit cognitive and metabolic health.

## Supplementary Information

Below is the link to the electronic supplementary material.

Supplementary file1 (PDF 248 KB)
