# Supplementary material for: Multi-night acoustic stimulation is associated with better sleep, amyloid dynamics, and memory in older adults with cognitive impairment
Source: GeroScience. 2024 May 14;46(6):6157–72. doi: 10.1007/s11357-024-01195-z (PMC11493878; doi:10.1007/s11357-024-01195-z)
Supplement: Supplementary file 1 — Supplementary file1 (PDF 248 KB) [file 11357_2024_1195_MOESM1_ESM.pdf]

GeroScience, Online Resource 1 (supplementary information) for

Multi-night acoustic stimulation is associated with better sleep, amyloid dynamics, and memory in older adults with cognitive impairment

Céline J. Zeller[1,2], Marina Wunderlin[1], Korian Wicki[1,2], Charlotte E. Teunissen[3], Christoph Nissen[4,5], Marc A. Züst*‡[1], Stefan Klöppel‡[1]

‡These authors contributed equally to this work.

[1] University Hospital of Old Age Psychiatry and Psychotherapy, University of Bern, 3000 Bern 60, Switzerland

[2] Graduate School for Health Sciences, University of Bern, 3012 Bern, Switzerland

[3] Neurochemistry Laboratory, Department of Clinical Chemistry, Amsterdam Neuroscience, Amsterdam UMC, Vrije Universiteit Amsterdam, 1081 HV Amsterdam, Netherlands

[4] Division of Psychiatric Specialties, Department of Psychiatry, Geneva University Hospitals (HUG), 1201 Geneva, Switzerland

[5] Department of Psychiatry, University of Geneva, 1201 Geneva, Switzerland

*Corresponding author:

Marc A. Züst

Email: marc.a.zuest@unibe.ch

Regression models of physiological response to stimulation on plasma Aβ pre- to post-intervention change scores: No effect of including covariates age and gender (model comparisons)

Table S1. PLAS-induced electrophysiological response predicts Amyloid Beta (Aβ) response to treatment

	All participants		Healthy		Cognitive impairment	
Model without covariates			**t-values**			
(intercept)	5.75	***	4.62	***	4.23	***
Aβ	0.71		-0.68		2.77	*
Model with covariates			**t-values**			
(intercept)	0.63		-0.47		0.86	
Aβ	0.67		-0.73		2.11	.
Age	-0.37		0.80		-0.40	
Gender	0.52		-0.42		-0.54	
Model comparison			**F-values**			
	0.20		0.61		0.26	

*** $p < 0.001$; ** $p < 0.01$; * $p < 0.05$; . $p = 0.058$. The stronger the physiological response to PLAS, the more favorable plasma amyloid levels developed from pre- to post-intervention in the cognitively impaired (CI)-group alone (last column). Age and gender did not explain changes in Aβ42/Aβ40 difference score (model comparison: $F(2, 11) = 0.26$, $p = 0.78$). Including these covariates in the regression model reduced the effect of the induced second SW peak to a strong trend ($p = 0.058$), probably due to overfitting*

Regression models of physiological response to stimulation on memory (performance in face-occupation associations (FOA) task): No effect of including covariates age and gender (model comparisons)

Table S2. PLAS-induced electrophysiological response and FOA-performance post-intervention

**Post-intervention**	All participants		Healthy		Cognitive impairment	
Model without covariates			**t-values**			
(intercept)	0.97		-0.18		1.46	
FOA	2.62	*	2.30	*	0.64	
Model with covariates			**t-values**			
(intercept)	0.26		-1.09		1.36	
FOA	2.44	*	2.15	.	0.93	
Age	-0.19		1.09		-1.30	
Gender	0.47		0.19		0.76	
Model comparison			**F-values**			
	0.14		0.60		1.33	

*** $p < 0.001$; ** $p < 0.01$; * $p < 0.05$; . $p < 0.1$

Table S3. PLAS-induced electrophysiological response and FOA-performance at follow-up 1

**Follow-up 1**	All participants		Healthy		Cognitive impairment	
Model without covariates			**t-values**			
(intercept)	1.82	.	-0.08		1.99	.
FOA	3.34	**	2.90	*	1.07	
Model with covariates			**t-values**			
(intercept)	0.16		-0.64		1.29	
FOA	3.11	**	2.51	*	0.76	
Age	0.00		0.60		-1.18	
Gender	0.32		0.39		0.30	
Model comparison			**F-values**			
	0.05		0.23		0.82	

*** $p < 0.001$; ** $p < 0.01$; * $p < 0.05$; . $p < 0.1$

Table S4. PLAS-induced electrophysiological response and FOA-performance at follow-up 2

**Follow-up 2**	All participants		Healthy		Cognitive impairment	
Model without covariates			**t-values**			
(intercept)	8.04	***	5.71	***	5.13	***
FOA	3.46	**	1.96	.	2.60	*
Model with covariates			**t-values**			
(intercept)	0.77		-0.38		1.37	
FOA	3.26	**	1.75		2.14	.
Age	-0.22		0.77		-1.06	
Gender	-0.40		-0.42		-0.14	
Model comparison			**F-values**			
	0.10		0.46		0.56	

**** $p < 0.001$; ** $p < 0.01$; * $p < 0.05$; . $p < 0.1$*

Including age and gender as covariates did not improve the models, nor did covariates associate with physiological response to stimulation (all model comparisons n.s, p>.30.; all covariates n.s., p>.22), but the main effect on memory performance (FOA) got weakened, probably due to overfitting.
